# Effect of Malignancy on Semen Parameters

**DOI:** 10.3390/life12060922

**Published:** 2022-06-20

**Authors:** Guy Shrem, Liat Azani, Ido Feferkorn, Tamar Listovsky, Sofia Hussaini, Benjamin Farber, Michael H. Dahan, Mali Salmon-Divon

**Affiliations:** 1 IVF Unit, Department of Obstetrics and Gynecology, Kaplan Medical Center, 1st Pasternak St., Rehovot 7661041, Israel; 2 Department of Molecular Biology, Ariel University, Ariel 40700, Israel; liat.azani@gmail.com (L.A.); tamary@ariel.ac.il (T.L.); malisa@ariel.ac.il (M.S.-D.); 3 Division of Reproductive Endocrinology and Infertility, McGill University Health Care Center, 888 Boul. de Maisonneuve E #200, Montreal, QC H2L 4S8, Canada; ido.feferkorn@mail.mcgill.ca (I.F.); sofia.hussaini@mail.mcgill.ca (S.H.); michael.dahan@mcgill.ca (M.H.D.); 4 Adelson School of Medicine, Ariel University, Ariel 40700, Israel; 5 Faculty of Medicine, McGill University, 3605 Rue de la Montagne, Montreal, QC H3G 2M1, Canada; ben.farber2000@gmail.com

**Keywords:** malignancy, semen parameters, sperm cryopreservation

## Abstract

Purpose: We aimed to examine how various types of cancer, classified histologically, affect semen quality. Methods: The study group included 313 patients who were diagnosed with cancer and reached for a sperm cryopreservation before a gonadotoxic treatment (PG-Tx group). Their semen parameters were compared to those of two control groups: (a) individuals who attended a fertility investigation and were found to be above the limit of the lower reference value of the WHO 2010 manual (ARL group), and (b) fertile men, whose semen parameters were obtained from the dataset of the WHO 2020 manual. Results: Semen quality was significantly poorer in the PG-Tx group than in the ARL group. Differences included a 65.6% decrease in concentration, a 12.1% decrease in volume, a 72.7% decrease in total count, and a 33.0%, 22.2%, and 24.7% decrease in total motility, rapid motility, and progressive motility, respectively. Linear regression models comparing the PG-Tx and ARL groups revealed that the maximum reduction in total motility and concentration was in men with germ-cell tumors, whereas the minimum reduction was in hematological tumors. Similarly, all sperm quality parameters were significantly lower in the PG-Tx group than in the fertile-men group (*p* < 0.0001). Conclusions: While the effect of malignancy on semen parameters is debatable, we found that all examined types of cancer significantly impaired sperm quality parameters. Although the median of most semen parameters of patients with cancer were still in the normal WHO range, their fifth percentile, represents men with a delayed time to pregnancy.

## 1. Introduction

The diagnosis of a malignant disease is a catastrophic life event at any age. For young individuals, malignancy-related concerns (such as treatment and prognosis) are often accompanied by concerns regarding the potential effects of the malignancy and treatment on future fertility. Due to the improvement in chemotherapy protocols and surgical techniques, the survival rate of individuals with cancer has increased substantially in recent years [1], further raising the concern regarding future fertility. Indeed, young patients should be counseled regarding the reproduction repercussions following cancer treatment. Advances in assisted-reproduction technologies and the cryopreservation of oocytes and sperm have emerged as an important option for these patients and referring individuals in reproductive ages to fertility-conservation procedures has become the standard of care [2]. Since anti-neoplastic treatments can be gonadotoxic, it is preferable to bank gametes prior to the first treatment of the malignancy [3]. For males, this usually includes the cryopreservation of ejaculated semen before the onset of gonadotoxic treatments. However, almost 25 years ago, it was reported [4] that the semen of oncologic patients is of lower quality, even before the first gonadotoxic treatment. The mechanism underlying this reduction in sperm quality in patients with cancer is still unclear and may involve stress, fever, or immune-mediated factors, and it may be related to cancer type [5].

Previous studies that investigated the effect of the type of malignancy on semen parameters indicate that Hodgkin’s lymphoma (HL) and testicular cancer (TC) have a deleterious effect on semen parameters [6,7,8,9]. Bizet et al. [10] found that patients with HL had significantly lower sperm concentrations and lower total sperm counts than patients with other malignancies; this effect may be attributed to fever, which can occur in HL, since hyperthermia can harm spermatogenesis [11,12]. Other possible explanations for the lower quality of semen in patients with HL are the direct damage to the germinal epithelium and the effect of cytokines, such as IL-1, IL-2, and tumor necrosis factor (TNF)-alpha on the hypothalamic-pituitary axis [13,14].

Several studies have confirmed the deleterious effect of TC on semen parameters [15,16,17]. Proposed mechanisms include the interruption of the blood–testicular barrier, the formation of anti-sperm antibodies, or the infiltration of lymphocytes into the parenchyma, adjacent to the tumor [18]. However, evidence regarding the effect of malignancy on semen parameters in other types of cancer is conflicting. Pallotti et al. [19] found that patients with non-Hodgkin’s lymphoma (NHL) were normozoospermic before treatment onset, whereas Williams et al. [20] demonstrated that men with various types of cancer (including HL, NHL, sarcoma, prostate cancer, leukemia, gastrointestinal cancer, central nervous system tumors, and unspecified or other rare malignancies) had pretreatment semen parameters in the fertile range for concentration and in the intermediate range for motility, whereas patients with TC demonstrated poorer semen parameters. Caponecchia et al. [8] found that the semen concentration in patients with TC or HL was lower than that in the fertile population, but such differences were not found for patients with other types of cancer. Conversely, several studies indicated that most patients with cancer demonstrate reduced sperm quality, irrespective of the type of cancer [notably, these studies also found significantly lower sperm concentrations in patients with TC, as compared with men with other types of cancer [21,22]. Amirjannati et al. [23] demonstrated that 71% of patients diagnosed with cancer (TC—59%, HL—15%, NHL—7%, other types of cancer—19%) had oligozoospermia, 93% had teratozoospermia, and 86% had asthenozoospermia. In addition, the authors reported that, whereas sperm concentrations were lower in the group of men with TC and HL, abnormal semen parameters accompanied all types of cancer.

In the current study, we have sought to examine the effect of different types of cancers on the quality of semen at the time of fertility preservation and before chemotherapy or radiation therapy. We employed a histological classification of cancer type—namely, classifying according to the tissue from which the cancer originated [24]—rather than classifying according to primary location, as different histological types of tumors may originate from the same location.

## 2. Materials and Methods

This is a retrospective cohort study. Our study group included 498 patients who attended the reproductive center of the McGill University Health Center (MUHC) in Montreal, Canada to preserve sperm due to a planned gonadotoxic treatment for cancer; these patients were classified as the pre-gonadotoxic treatment (PG-Tx) group. Only the first collected specimen for each patient was included in the analysis. None of these patients had received chemotherapy or radiation therapy before preserving the sperm specimen. Due to the intention to freeze the entire specimen, no sperm morphology assessment was performed, as this would have eliminated some of the cells due to fixation. Therefore, this parameter could not be compared to the control groups (see below). Patients who were exposed to a gonadotoxic treatment before sperm banking, patients who planned a gonadotoxic treatment due to a disease other than cancer, and patients with rare cancer represented by fewer than 25 samples in our cohort were excluded from the study. The final study group after exclusion thus included 313 patients.

The parameters of semen from the above-mentioned patients were compared to those of a control group, which included individuals without any history of cancer or gonadotoxic treatment, and who attended the MUHC for a fertility investigation between January 2009 and December 2018. As described in our previous study [25], a total of 17,915 samples were collected during this period. After limiting the analysis to one sample per patient and including only samples that surpassed the lower reference limits set for normal semen parameters by the 2010 WHO [concentration >15 mil/mL, motility ≥40%, morphology >4%] [26] we left with 6282 control samples. These samples were classified as the above reference limit (ARL) group. The age distribution of the PG-Tx and ARL cohorts is presented in Supplementary File S1.

Since the samples in the ARL group were obtained from individuals who were referred to a fertility clinic, they likely do not represent the general fertile population. To overcome this limitation and to estimate the difference in semen parameters between patients with cancer and fertile individuals, a comparison was also conducted to a fertile cohort. We used data from the trials that defined the reference ranges for the WHO manual (6th edition), collected over the past three decades and available at https://doi.org/10.15132/10000163, accessed on 17 February 2022 [27]. The samples in this dataset were taken from individuals from 12 countries and five continents, with a time to pregnancy of up to and including 12 months and with a defined abstinence period of 2–7 days.

Finally, we compared between the subgroups of cancer, as classified according to cancer histology based on the National Institutes of Health (NIH) classification [24]. The types of cancers included in each group are listed in Supplementary File S1. After grouping the hematological cancers due to relatively small numbers of each type, and after excluding CNS tumors due to an overall small number of patients in the database (*n* = 18), we compared four subgroups: hematological tumors (leukemia and lymphoma), germ-cell tumors, carcinoma, and sarcoma. Ethics approval was obtained through the Institutional Review Board (IRB) and the Institutional Ethics Committee of the MUHC number 2020–5643.

### Statistical Analyses

Descriptive statistics are presented as number (*n*), mean ± standard deviation (SD), or median and box plots, as appropriate. The *p* values of differences between two groups were calculated by the Mann–Whitney U test, and for multiple groups by the Kruskal–Wallis rank-sum test, followed by a Dunn’s post hoc test [28]. Multiple linear regression analyses, adjusted to age, were used to assess the influence of cancer on semen parameters. Due to violation of the residual normality assumption of some of the semen parameters, we performed a log transformation of the total count, concentration, and volume parameters before the linear regression analysis. To interpret the coefficients observed in the regression model of the transformed parameters, we used the formula (exp(coefficient) − 1) × 100, yielding the percent difference (increase or decrease) in the response of the cancer group compared with the reference group. To quantify the impact of cancer on semen parameters, we conducted a multiple linear regression using the group (ARL vs. PG-Tx) as a predictor and adjusting for age (Table 1). Table 1 and Table 2 present the original coefficient for non-transformed semen parameters and the percent difference for the transformed ones. All calculations and figures were generated with the R software environment (version 4.1.1). The pie chart was generated using Pie Chart Maker [29].

## 3. Results

**Figure 1:**
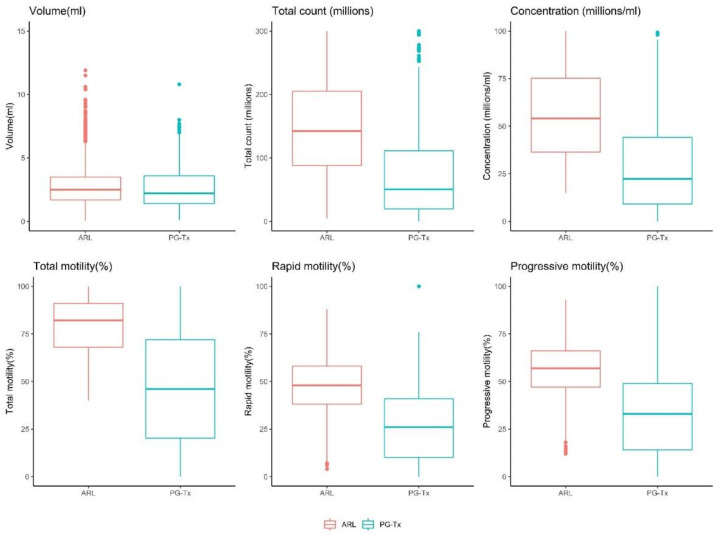
Distribution of semen parameters across the ARL and PG-Tx groups. *p* < 0.0001 for all comparisons except for volume for which *p* < 0.01 (Wilcoxon test).

The total sperm count, motility (%), rapid (%), progressive motility (%), concentration, and volume (ml) were significantly lower in the PG-Tx group than in the ARL group (Figure 1). Cancer and semen parameters were significantly and negatively correlated (Table 1) such that, on average, samples from patients with a diagnosis of cancer were associated with a 65.6% decrease in concentration, a 12.1% decrease in volume, a 72.7% decrease in total count, and a 33.0%, 22.2%, and 24.7% decrease in total motility, rapid motility, and progressive motility, respectively.

**Figure 2:**
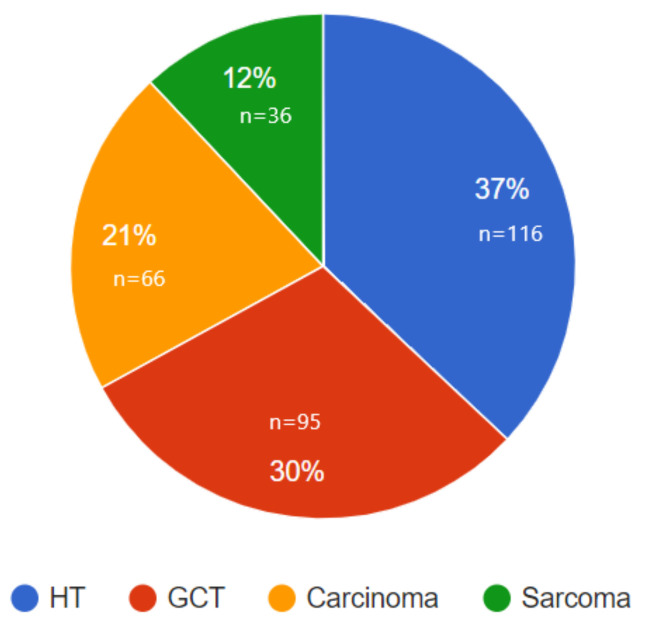
Distribution of tumors in the PG-Tx group according to histological type (% of 313 samples in total).

**Figure 3:**
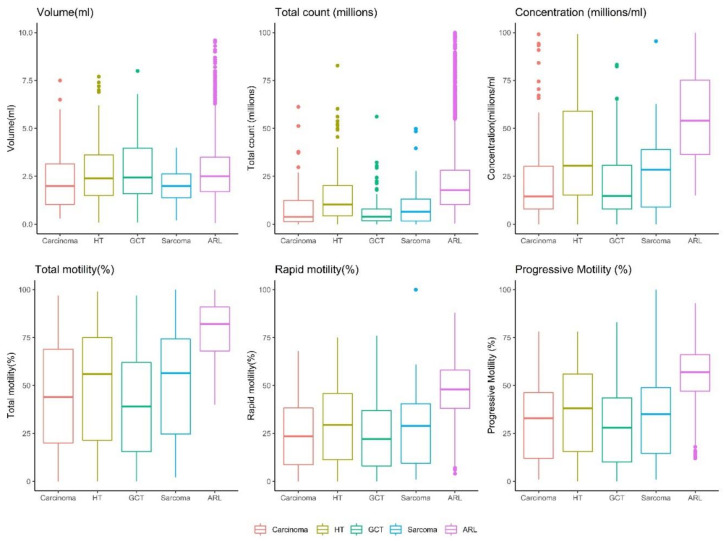
Distribution of semen parameters across tumor subgroups. HT = hematological tumors, GCT = germ cell tumors. *p* < 0.0001 for all comparisons except for volume for which *p* < 0.01 (Kruskal–Wallis test).

A comparison between the various histological cancer types is presented in Supplementary File S2 and in Figure 2 and Figure 3. Most samples were hematological tumors (leukemia and lymphoma, 37%), followed by germ-cell tumors (30%), carcinoma (21%), and sarcoma (12%) (Figure 2). The descriptive statistics of each group (Supplementary File S2) indicate that the median sperm parameters for each cancer type, except for germ-cell tumors, were within the normal WHO 2010 range. For patients with germ-cell tumors, the median total motility (39%) and the median concentration (14.9 mil/mL) were slightly lower than the limit range.

**Figure 4:**
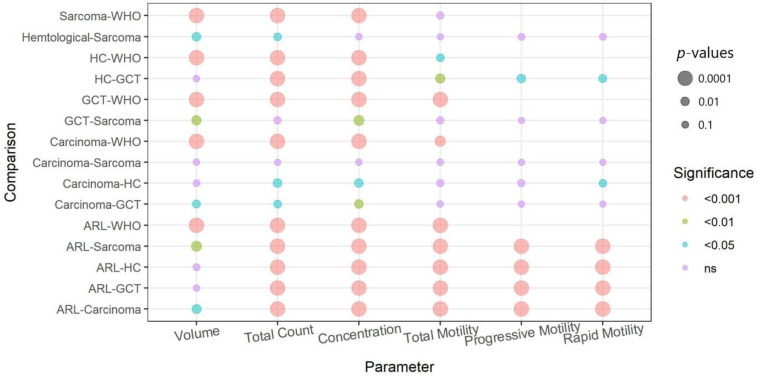
*p*-values of the different comparisons (Dunn’s post hoc test). Circle sizes and colors are proportional to the *p* value. HT = hematological tumors, GCT = germ cell tumors.

Except for volume, all the semen parameters of the ARL group were significantly higher than those of the patients with cancer, regardless of cancer histological type (*p* < 0.0001, Figure 4). For volume, the ARL group values were significantly higher only compared with patients with sarcoma or carcinoma (*p* < 0.001, Figure 4). The semen parameters not only differed between the ARL and the PG-Tx groups, but they also differed between patients with different cancer types (Figure 3, and see *p* values in Figure 4). The total sperm count of patients with hematological tumors was significantly higher than that of patients with other types of tumor, and the concentration was higher in these patients than in patients with carcinoma or germ-cell tumors. Sperm motility was higher in patients with hematological tumors than in patients with germ-cell tumors and the semen volume of patients with hematological or germ-cell tumors was significantly higher than that in patients with sarcoma (Figure 3 and Figure 4).

To further estimate the effect of the cancer subgroup on semen parameters, we performed linear regression models, in which we included the cancer subgroup as an independent predictor while adjusting for age (Table 2). As compared with the ARL group, the maximal reduction in total motility was found in patients with germ-cell tumors (−38.7%) and the minimum reduction was found in patients with hematologic tumors (−28.7%). Similarly, a maximal reduction in concentration was detected in patients with germ-cell tumors (78.8% lower than the ARL group) and the minimum reduction was found in patients with hematologic tumors (48.5% lower than the ARL group).

**Figure 5:**
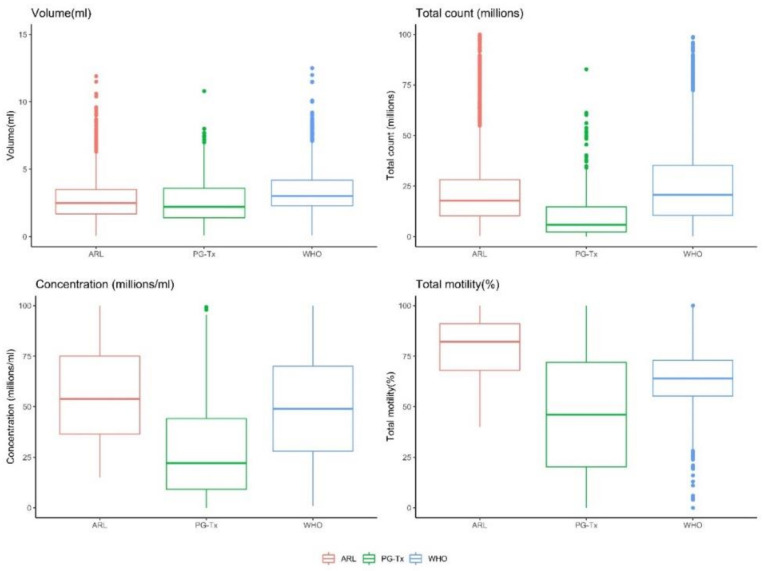
Distribution of semen parameters across the WHO fertile cohort, the ARL group, and the PG-Tx group. *p* < 0.0001 for all comparisons (Kruskal–Wallis test).

**Figure 6:**
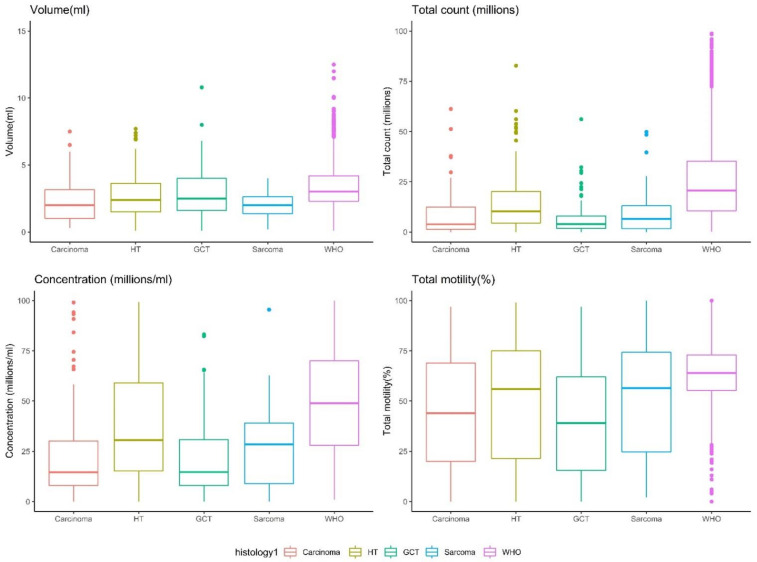
Distribution of semen parameters across the WHO group and the tumor subgroups. HT = hematological tumors, GCT = germ cell tumors. *p* < 0.0001 for all comparisons (Kruskal–Wallis test).

In comparison to the WHO group of fertile men (Figure 5), all four tested parameters (volume, total count, concentration, and total motility) were significantly lower in the PG-Tx group (*p* < 0.0001). Specifically, regarding semen volume, the differences between the PG-Tx and the ARL groups were minor (albeit significant; *p* < 0.01), but the differences between men in the PG-Tx group and fertile men in the WHO dataset were much greater (*p* < 0.0001). Finally, a separate comparison between the semen parameters of men in the WHO group and those of men with each type of tumor (Figure 6, and see *p* values in Figure 4) revealed that the total count, concentration, and volume of men in the WHO group were significantly higher than those of men with cancer, regardless of the cancer histological type (*p* < 0.0001). The total motility in the WHO group was significantly higher than that of all cancer subtypes except sarcoma (*p* < 0.001 for carcinoma and germ-cell tumors, *p* < 0.05 for hematological tumors). Importantly, a wide distribution was observed in sperm motility in all cancer types, as compared with motility in men from either the WHO or the ARL groups.

## 4. Discussion

Because the number of patients with cancer referred for fertility preservation is increasing [30], it is important to understand the effect of cancer histology on semen parameters. We present a comprehensive study to compare the semen parameters of individuals from three groups: patients who performed sperm cryopreservation before a planned gonadotoxic treatment due to recently diagnosed cancer (the PG-Tx group); a cohort of patients who attended our clinic for fertility investigation and were found to have normal sperm parameters according to the 2010 WHO reference limits (the ARL group); and a cohort from a database representing healthy fertile males (WHO 2020). The PG-Tx group was further divided based on cancer histology.

The pathophysiology of the deleterious effect of cancer on semen parameters is not fully understood. In germ cell cancer it has been proposed that the impairment is associated with the effect of the tumor and its hormonal secretion such as beta-hCG that interfere with spermatogenesis [31]. Hematological cancer comes with prolonged fever that might affect spermatogenesis [14]. Having cancer disease in general may lead to stress that as well might affect spermatogenesis [32].

We found a significant negative association between cancer and semen parameters and, in agreement with previous studies [8,10,19], we found that the median sperm parameters for each cancer type were within the normal WHO range—except for germ-cell tumors, which demonstrated slightly lower median sperm parameters. However, the entire distribution in all parameters was shifted toward lower sperm quality values (for example, while in the WHO group only 5% of the samples had a motility below 40%, it increased to 44.7% in the PG-Tx group).

Our findings are different from those of Caponecchia et al. [8], who found no difference in sperm parameters between patients with various types of cancer (all treated as a single group) and healthy men. Pallotti et al. [19] found that most subjects with NHL were normozoospermic. We did not investigate this group by itself but, rather, included it in the hematologic cancer group, along with HL and leukemia, which may explain the decrease in semen parameters that we observed in this group.

Comparing the distribution of different semen parameters across different tumor subgroups revealed that the individuals with germ-cell tumors had a significantly lower sperm concentration than those with any other tumor type. This finding is concomitant with previous reports, which indicated that patients with TC have the lowest sperm concentrations of all oncology categories [33,34]. This is probably because germ-cell tumors are prone to disrupt the entire process of semen production and function [15]. Several other studies have also found that germ-cell tumors have a detrimental effect on semen parameters, as compared with HL cancers [31,35]. We also found a significant difference in all tested parameters (except volume) between patients with germ-cell tumors and those with hematological tumors. Since sperm volume may be affected by several factors—including psychological factors that are prominent in recently diagnosed cancer patients—the volume comparison results should be treated with caution.

Importantly, our cohort does not reflect the reported incidence of cancer in the general population. For example, whereas the incidence of NHL and HL in the general population is only 3.3% and 0.5%, respectively [24], this group comprised 30% of males who froze sperm in our cohort. Since young males in the reproductive age are those who are mainly referred to sperm cryopreservation by the referral clinics, our cohort is enriched with tumor types that are more common in the young population, such as HL and germ-cell tumors.

The limitations of our study include the absence of morphological parameters that could complete the analysis of semen parameters and the lack of data related to tumor stage and grade, which were unavailable to us. The need to group different types of cancer based on histology prevented an in-depth comparison of each tumor type separately. On the other hand, it allows for a more homogenous grouping than that used in previously published studies. Moreover, to diminish bias in sample analysis, our study compared tumor groups, not only to the ARL group from the same institution, but also to the healthy fertile male population, represented by the WHO 2020 data.

Perhaps most importantly, our study assesses semen parameters as a surrogate for a more important factor: fertility. While individual semen parameters are considered poor predictors of overall male fertility [36,37,38] multiple studies have shown that they may help estimate the length of time needed to achieve pregnancy [39,40,41]. For example, a large European study showed that a sperm concentration up to 55 million and a total sperm count of up to 145 million, which are well beyond the WHO reference levels, are associated with an earlier time to pregnancy in fertile couples [40]. Another study showed that a higher sperm count, concentration, and progressive motility beyond the WHO references are associated with better conception rates and time to conception [42].

Overall, our findings suggest that various types of cancer significantly impair sperm parameters and that, although most parameters are still within the normal WHO range, the fifth percentile represents men with a delayed time to pregnancy. The specific effect of this deterioration on the fertility of patients with cancer needs to be further examined in future follow-up surveys.

## Figures and Tables

**Table 1:** Effect of cancer on semen parameters after adjusting to age.

	Beta	95% CI	*p*	R^2^
Volume *	−12.1%	−16.3%, −8.6%	<0.001	0.02
Concentration *	−65.6%	−68.3%, −62.8%	<0.001	0.10
Total count (millions) *	−72.7%	−75.0%, −69.0%	<0.001	0.09
Total motility (%)	−33	−34.9, −31.1	<0.001	0.15
Rapid motility (%)	−22.2	−23.8, −20.6	<0.001	0.10
Progressive Motility (%)	−24.7	−26.3, −23.1	<0.001	0.09

* These parameters were log-transformed before the regression analysis. The coefficient shown for these parameters is the percent difference (a positive number indicates an increase, and a negative number indicates a decrease) of the PG-Tx group compared with the ARL group. For non-transformed parameters, the original coefficient is shown.

**Table 2:** Effect of cancer subgroup on semen parameters, using ARL as the reference group, after adjusting to age.

Semen Parameter	Cancer Type	Beta	95% CI	*p*
Total count (millions) *	Carcinoma	−73.3%	−78.3%, −67.2%	<0.001
	Hematological cancer	−57.6%	−63.8%, −50.4%	<0.001
	Germ cell tumor	−82.7%	−85.4%, −79.4%	<0.001
	Sarcoma	−76.8%	−82.4%, −69.3%	<0.001
Volume (mL) *	Carcinoma	−11.6%	−19.4%, −3.11%	<0.01
	Hematological cancer	−12.4%	−18.4%, −5.9%	<0.001
	Germ cell tumor	−7.4%	−14.3%, −0.007%	<0.05
	Sarcoma	−27.3%	−35.9%, −17.6%	<0.001
Concentration (mil/mL) *	Carcinoma	−69.4%	−74%, −64.1%	<0.001
	Hematological cancer	−48.5%	−54.5%, −41.7%	<0.001
	Germ cell tumor	−78.8%	−81.5%, −75.7%	<0.001
	Sarcoma	−57.9%	−66.2%, −47.5%	<0.001
Total motility (%)	Carcinoma	−33.3	−37.2, −29.4	<0.001
	Hematological cancer	−28.7	−31.7, −25.7%	<0.001
	Germ cell tumor	−38.7	−42, −35.4	<0.001
	Sarcoma	−31.2	−36.5, −25.8	<0.001
Rapid motility(%)	Carcinoma	−22.5	−25.9, −19.2	<0.001
	Hematological cancer	−19.8	−22.3, −17.2	<0.001
	Germ cell tumor	−24.4	−27.3,−21.6	<0.001
	Sarcoma	−23.4	−27.9,−18.9	<0.001
Progressive Motility (%)	Carcinoma	−24.4	−27.8, −21	<0.001
	Hematological cancer	−21.7	−24.3, −19.1	<0.001
	Germ cell tumor	−28.1	−31, −25.2	<0.001
	Sarcoma	−26.2	−30.8, −21.6	<0.001

* These parameters were log-transformed before the regression analysis. The coefficient shown for these parameters is the percent difference (a positive number indicates an increase, and a negative number indicates a decrease) of the cancer subgroup, as compared with the ARL group. For non-transformed parameters, the original coefficient is shown.

## Supplementary Materials

The following supporting information can be downloaded at: https://www.mdpi.com/article/10.3390/life12060922/s1, File S1: Cohort age distribution and types of cancers included in the study; File S2: Descriptive statistics of semen parameters.
